# Dimensional Stability and Process Capability of an Industrial Component Injected with Recycled Polypropylene

**DOI:** 10.3390/polym11061063

**Published:** 2019-06-20

**Authors:** José Eduardo Galve, Daniel Elduque, Carmelo Pina, Isabel Clavería, Raquel Acero, Ángel Fernández, Carlos Javierre

**Affiliations:** 1 BSH Electrodomésticos España, S.A., Avda. de la Industria, 49, 50016 Zaragoza, Spain; jose.galve@bshg.com (J.E.G.); carmelo.pina@bshg.com (C.P.); 2 i+AITIIP, Department of Mechanical Engineering, University of Zaragoza EINA, Maria de Luna 3, 50018 Zaragoza, Spain; delduque@unizar.es (D.E.); afernan@unizar.es (Á.F.); carlos.javierre@unizar.es (C.J.); 3 Department of Design and Manufacturing Engineering, University of Zaragoza EINA, Maria de Luna 3, 50018 Zaragoza, Spain; racero@unizar.es

**Keywords:** polypropylene, dimensional, injection, recycled, process capability, warpage, shrinkage

## Abstract

The usage of recycled polymers for industrial purposes arises as one of the most promising methods of reducing environmental impact and costs associated with scrapping parts. This paper presents a systematic study of the dimensional stability of a raw and 100% recycled polypropylene subjected to realistic environmental conditions occurring along its working life. The component studied is an internal part of an induction hob assembly. Industrial samples manufactured with both materials, in the same mold, and in the same injection machine, are subjected to ejection conditions, storage conditions (50 °C), and extreme performance conditions (80 °C). Induced dimensional changes are registered and analyzed using a coordinate measuring machine, and a tactile sensing probe. To verify the process capability of the samples manufacturing, *C*_p_ and *C*_pk_ values are calculated to evaluate the suitability of the recycled material as an alternative. Results conclude that, although the use of recycled material implies slight differences in terms of dimensional stability due to the changes induced in the polymer structure, these differences are not significant enough to affect the injection process capability. Therefore, recycling arises as one effective method to reduce both overruns associated with the consumption of raw polypropylene material and its environmental impact.

## 1. Introduction

The annual growth in polypropylene (PP) consumption has been increasing again during the last years, after the severe decline suffered ten years ago due to the economic crisis [1].

This new trend leads to higher prices imposed by producers as well as higher amounts of waste due to plastic cuts and scraps that result in significant overruns. On the other hand, waste material may follow different end-of-life treatments, from which landfilling or incineration cause the highest environmental impact [2]. At this point, recycling stands out as a much more environmental-friendly alternative end-of-life treatment for polymers as it has also been promoted by specific key actions by the European Union [3,4]. One of the approaches to sustainability that has been gaining momentum in recent years is the circular economy, which encourages industries to convert waste back to materials through recycling [5,6,7,8,9]. Other strategies working on the same idea are reusing, reparability, refurbishment, and remanufacturing [10].

Research shows that the environmental impact of products can be highly reduced by manufacturing with recycled polymers instead of raw materials [11,12,13]. Recycled material can be mainly obtained by chemical or mechanical means [14,15,16,17,18]. Aiming for the same goals, these processes differ significantly. While chemical recycling uses organic solvents that may be dangerous to the environment [19], mechanical recycling is referred to operations that aim to recover plastic waste via mechanical processes (i.e., grinding, washing, separating, drying, re-granulating, or compounding) causing the break of polymer chains of the structure of PP [20]. As a side effect to consider, we should mention contamination given that, during the recycling process, it can also deteriorate the polymers [21], and whose alterations lead to changes in both mechanical and rheological properties [22,23].

The injection molding process is affected by rheological behavior, especially at the filling stage [24,25]. However, it is not the only process affected by the replacement of raw materials with recycled ones. In fact, as molding and processing are designed for a specific material, problems related to warpage can arise when the material is changed, and process parameters could be adjusted to meet the part requirements [26].

Polymer parts suffer from shrinkage and warpage during the injection molding process and induced by thermal and pressure changes achieved over it, especially those injected with semi-crystalline materials like PP, [27]. As a result, the final dimensions of injected components are affected by material shrinkage during the process caused by the filling orientation, packing conditions, and cooling parameters [28,29]. Different models have been proposed to study this effect [30,31] as this issue is important because it directly affects the functionality of the part. Therefore, many different authors take it into account when a modification on the material is done. Khanjanzadeh et al. analyzed the dimensional stability of replacing raw PP with recycled PP in the manufacturing of wood-plastic composites [32]. De Carvalho et al. also reported the influence of mold material on the dimensional stability [33]; and Norihisa et al. analyzed the dimensional stability of different PP wood composites [34]. Dimensional stability has also been increased through assisted-injection molding processes such as gas assisted injection molding [35,36].

Induced warpage on the part, due to changes in material, leads to problems in keeping the part dimensions within the specifications [37], which is required to avoid later assembly operations. When the material is changed, a new mold should be provided with the shrinkage requirements of the new material. In order to avoid the cost of a new mold and achieve the dimensional requirements of the product, an optimization of the process conditions with the recycled material is suggested Along with its working life and during the period in-between, its manufacture and the beginning of its working life, a component can be subjected to different environmental conditions that can affect its dimensional state [38]. In this paper, the effect of using a recycled material instead of a raw one on the dimensional stability of the parts is studied. While previous researches have focused on the cause of dimensional distortions such as shrinkage and warpage caused by process conditions [39,40], hardly any deal with the topic of dimensional stability of injected components when the material is replaced by a recycled one, or along its working life due to changes in the environmental conditions.

The main contribution of this paper consists of a systematic investigation into the dimensional changes of raw and recycled PP intended for long-term use under different realistic in-service environmental conditions to which the component is subjected during its working life. The paper analyzes not only the dimensional changes but also the manufacturing process capability according to *C*_p_ and *C*_pk_ parameters [41,42]. C_p_ stands for process capability to produce parts within the tolerance specification limits and *C*_pk_ stands for process capability to produce parts within the tolerance specification limits and near its nominal value.

The following section of this paper explains the materials used and the dimensional and process capability test methodologies. Then, the dimensional results are shown under different conditions (after stabilization, after storage, and after extreme performance), and the dimensional behavior is analyzed, followed by the analysis of the process capability parameters. Finally, conclusions are obtained after analyzing the results.

## 2. Materials and Methods

### 2.1. Materials

Two PP materials filled with 40% of talc are used for the tests, a raw PP Hostacom HBC 309 NAT, and a 100% recycled one, E-RIALFILL H 07 40 T (Table 1). The recycled one is a polymer obtained from post-industrial PP waste materials, tracked to ensure that only pure propylene free of impurities is used.

**Figure 1:**
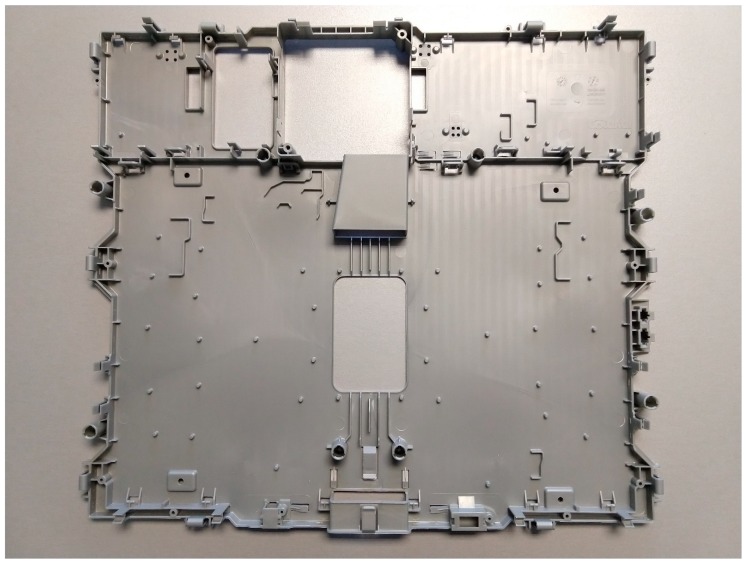
Sample component used for the analysis.

The component selected for the study is an internal part of an induction hob assembly that must meet several dimensional requirements to fit with other parts of the induction hob (Figure 1). The overall nominal dimensions are 472.6 mm × 534 mm and its nominal thickness is 1.8 mm.

### 2.2. Dimensional Tests Methodology

Sample components were obtained by an injection mold in a Negri Bossi 8000H-6700 (Negri-Bossi, Cologno Monzese, Italy) injection molding machine [43], at a melting temperature of 240 °C, injection time of 4.5 s, packing pressure of 30 MPa for 2 s, and a further cooling time of 20 s. The same injection parameters were used for both materials. All the samples were injected on the same day and with the same mold after the stabilization of the process.

Fifteen samples of each material were measured under three different temperature conditions with the same exposure time of 48 h. A preliminary test, named as *M*_0_, was conducted after having the stabilized parts under lab conditions at a room temperature of 20 ± 0.5 °C. This setting represents the average standard temperature conditions of the component just after having been injected. Since *M*_0_ is the absolute dimension measured during the test, ∆*M*_0_ is the dimension measurement referenced to nominal dimension (*M*_nom_) calculated as:

(1) $$\Delta M_{0}=\left|M_{0}-M_{\mathrm{nom}}\right|,$$

Then, a second test named as *M*_1_ was conducted by introducing the samples into a Memmert CTC256 environmental test chamber, (Memmert, Schwabach, Germany), at a temperature of 50 °C for 48 h. After this time, samples were stabilized at 20 °C for 24 h [44]. This second test represents a medium/high-temperature condition storage situation, similar to those found in the place where these parts are stored for some time before they are ready to be assembled, which is the most critical situation in the assembly chain regarding temperature. 50 °C is the maximum temperature that the stored components reach during summer. Measurement values registered under these conditions were named as *M*_1_. The difference in dimensions between the samples at 50 °C and the samples at room temperature, $\Delta M_{1}$, both referred to *M*_nom_, is calculated from the Equation (2):

(2) $$\Delta M_{1}=│\left|M_{0}-M_{\mathrm{nom}}\right|-\left|M_{1}-M_{\mathrm{nom}}\right|│,$$

where

M_1_ = dimension measured after being heated at 50 °C
*M*_nom_ = nominal dimension
*M*_0_ = dimension measured at room temperature
∆*M*_1_ = dimension variation between samples heated at 50 °C and samples at room temperature.

In order to reproduce temperature variations that take place on the samples performing their function into the assembly, reaching extreme performance temperatures close to 80 °C and cooling them to a standard temperature later, they were again heated up to 80 °C for 48 h and, then, stabilized under lab conditions of 20 ± 0.5 °C for 24 h [44]. This maximum performance temperature of 80 °C is the highest temperature that the part reaches. It has been measured with thermocouples after running an induction hob for several hours under stress conditions. Results of this third test were named as *M*_2_. The difference in dimensions between samples at 80 °C and at room temperature, $\Delta M_{2}$, both referred to *M*_nom_, is calculated from Equation (3):

(3) $$\Delta M_{2}=│\left|M_{0}-M_{\mathrm{nom}}\right|-\left|M_{2}-M_{\mathrm{nom}}\right|│,$$

where

M_2_ = dimension measured after being heated at 80 °C
*M*_nom_ = nominal dimension
*M*_0_ = dimension measured at room temperature
∆*M*_2_ = dimension variation between samples heated at 80 °C and at room temperature.

Shrinkage behavior of the samples between two different temperatures above room temperature, 50 and 80 °C, is also calculated from Equation (4):

(4) $$\Delta M_{50-80}=\left|\Delta M_{2}-\Delta M_{1}\right|,$$

where,

∆*M*_50-80_ is the difference in measures between samples heated at 50 and 80 °C.
∆*M*_i_ is the difference in measures between the dimensions at *M*_i_ conditions and those at room temperature; both referred to the nominal dimension.
*M*_1_ = dimension measured after being heated at 50 °C
*M*_2_ = dimension measured after being heated at 80 °C

**Figure 2:**
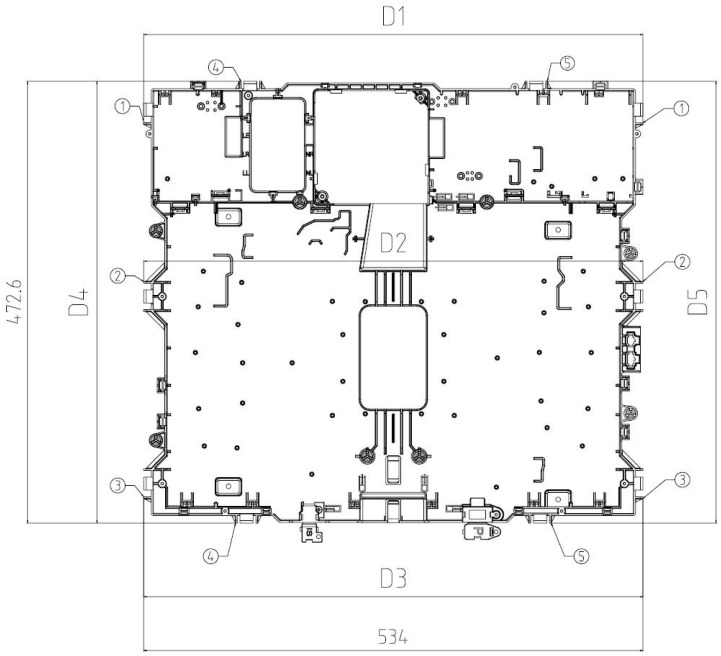
Detail of the locating areas of the part from which dimensions Di (mm) are defined.

Dimensions that will be analyzed at *M*_0_, *M*_1_, and *M*_2_ conditions are critical in terms of assembly and functionality. They are the ones locating part edges on the assembly frame, as described in Figure 2.

- D1 is the cartesian distance from fixing point 1 on the left edge of the part to fixing point 1 on the right edge. To determine the measurement of this distance, the line joining fixing points 1 and 3 is taken as a reference in such a way that D1 is normal to it. D1 nominal value is 534 ± 0.6 mm.
- D2 is the cartesian distance from fixing point 2 on the left edge of the part to fixing point 2 on the right edge. To determine the measurement of this distance, the line joining fixing points 1 and 3 is taken as a reference in such a way that D2 is normal to it. D2 nominal value is 534 ± 0.6 mm.
- D3 is the cartesian distance from fixing point 3 on the left edge of the part to fixing point 3 on the right edge. To determine the measurement of this distance, the line joining fixing points 1 and 3 is taken as a reference in such a way that D3 is normal to it. D3 nominal value is 534 ± 0.6 mm.
- D4 is the cartesian distance from fixing point 4 on the upper edge of the part to fixing point 4 on the lower edge. To determine the measurement of this distance, the line joining fixing points 4 and 5 is taken as a reference in such a way that D4 is normal to it. D4 nominal value is 472.6 ± 0.6 mm.
- D5 is the cartesian distance from fixing point 5 on the upper edge of the part to fixing point 5 on the lower edge. To determine the measurement of this distance, the line joining fixing points 4 and 5 is taken as a reference in such a way that D5 is normal to it. D5 nominal value is 472.6 ± 0.6 mm.

**Figure 3:**
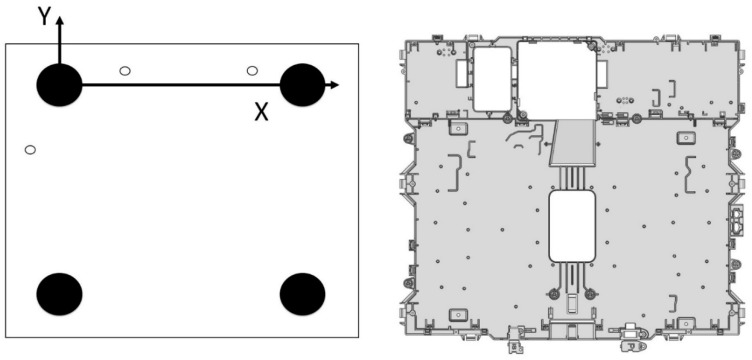
Base reference system to locate samples into a measurement tool.

To locate the component and record measurements data, a Zeiss PMC 876, (Carl Zeiss, Oberkochen, Germany) coordinate measuring machine was used [45]. The tactile scanning probe used was a Vast XT (Carl Zeiss, Oberkochen, Germany), [46], and the metrology software was Calypso (Carl Zeiss, Oberkochen, Germany) [47]. The base reference system was placed in the locating tool to automatize measurements from this baseline once the tool was in the measuring machine [44]. Figure 3 shows this base reference system. The component was always supported on the tool in the same way: first on the XY plane, then on the XZ plane, and finally, on the YZ plane to ensure that the component location was repeatable.

### 2.3. Process Capability Tests Methodology

To examine the suitability of recycled PP for the injected component from the point of view of quality controls during manufacturing, process capability is analyzed for both raw and recycled materials. Twenty-five new samples of each material were obtained by injection molding in the same conditions as described in the previous section.

Two representative dimensions of the width and height of the component (D1 and D4) were selected according to the critical assembly criteria to analyze the process capability. D1 and D4 were measured in the Zeiss PMC 876 coordinate measuring machine (Carl Zeiss, Oberkochen, Germany). It has been checked that measurements followed a normal distribution using the Anderson−Darling normality test. The reference system used was the same as the one used for dimensional tests, described in Figure 3. Process capability is calculated from parameters *C*_p_ and *C*_pk_, which are obtained from Equations (5) and (6). Low *C*_p_ values are related to high variability with respect to the specification values, whereas low *C*_pk_ values imply that the process is not centered between the specification limits.

(5) $$C_{p}=\frac{\left|\mathrm{USL}-\mathrm{LSL}\right|}{6\sigma },$$

(6) $$C_{\mathrm{pk}}=\min\left(C_{\mathrm{pu}},\;C_{\mathrm{pl}}\right)=\min\left(\frac{\left|\mathrm{USL}-\mu \right|}{3\sigma },\frac{\left|\mu -\mathrm{LSL}\right|}{3\sigma }\right),$$

where,

*C*_p_ stands for process capability to produce parts within the tolerance specification limits,
*C*_pk_ stands for process capability to produce parts within the tolerance specification limits and near its nominal value,
*C*_pu_ stands for the value between the process mean and the upper specification limit,
*C*_pl_ stands for the value between the process mean and the lower specification limit,
USL is the upper specification limit,
LSL is the lower specification limit.
Provided that measurements follow a normal distribution,
σ is the standard deviation of the measures
μ is the average value of n measured samples x_i_ calculated as:
  (7) $$\mu =\frac{\sum_{i=1}^{n}x_{i}}{n},$$
  where n = 25;
x_i_ is the value of each dimension for sample i.

## 3. Results and Discussion

### 3.1. Dimensions after Stabilization at Room Temperature

Table 2 shows dimension measurements for all the samples after stabilization at room temperature *M*_0_, and ∆*M*_0_, referenced to nominal temperature and calculated according to the Equation (1).

All the dimensions reduce their values regarding their nominal values after stabilization at 20 °C due, mainly, to the shrinkage induced by the temperature difference between the temperature at the ejection time, and the temperature at measurement time after stabilization. During the injection molding process, shrinkage is mainly influenced by three factors: (i) the holding pressure on the compressible polymer material during the packing stage, (ii) the flow orientation during the filling stage, and (iii) the temperature difference during the cooling stage [28,29]. All these effects lead to a global volumetric shrinkage of the component which remains constrained into the mold up to the ejection stage. Once the part is ejected, it keeps cooling until reaching room temperature (20 °C). During this time, the effect of temperature difference remains active provoking the shrinkage of the component and, thus, a decreasing of its dimensions.

**Figure 4:**
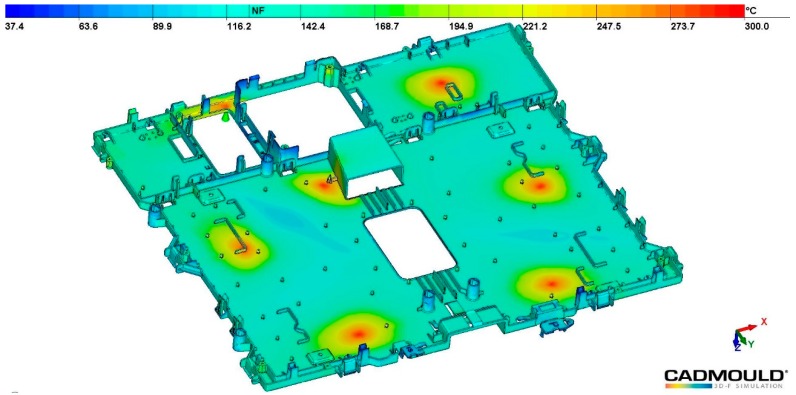
Temperature distribution on the sample at the ejection time.

Dimensions reduction achieved in samples with recycled material are slightly higher for all dimensions. Ejection temperature is slightly higher for recycled material (118 °C) than raw ones (110 °C). When samples are ejected at their respective ejection temperatures and, then, freely cooled down to the room temperature (20 °C), the thermal difference is higher for recycled material than for the raw one, contributing to a higher shrinkage and, thus, higher ∆*M*_0_ values in samples manufactured with recycled material. Although D1, D2, and D3 come from the same nominal value, they do not reach the same final dimensions. The lowest value is achieved for D1 and the highest for D3, being ∆*M*_0_ 58% higher in this case. These differences can be attributed to a differential shrinkage in different areas of the part sample caused by a differential cooling, and to different stiffness in the different part sections. Differential cooling is caused by non-uniform thickness in the sample section or by the design of the cooling system of the mold that is not capable of assuring a uniform cooling temperature along the sample surface. Figure 4 shows the temperature distribution at the ejection time. Most of the part surface presents a temperature around 110–120 °C. However, there are other surfaces marked as blue (60 °C) in which the temperature is lower because they are perpendicular to cooling channels and, in this case, heating extraction from the polymer core is less effective. Other surfaces that present a different temperature are the ones near the injection points, marked in yellow (165 °C), which are hotter because they are close to the polymer entrance to the mold. Sample stiffness can also vary in different sample sections where Di are measured because of the geometry features occurring in these sections. In the case of D1, the section is featured with a set of holes that makes the cross-section less stiff, leading to a higher deformation of the part. A cross-section in which D3 is measured lacks any hole, and it remains stiffer leading to a lower part deformation and a lower ∆*M*_0_. D4 and D5 come from the same nominal value, but the D4 dimensional variation is higher than the D5 also due to a lower stiffness in the D4 hollowed cross-section, which leads to a higher deformation.

### 3.2. Dimensions after Storage, and Extreme Performance Conditions

**Figure 5:**
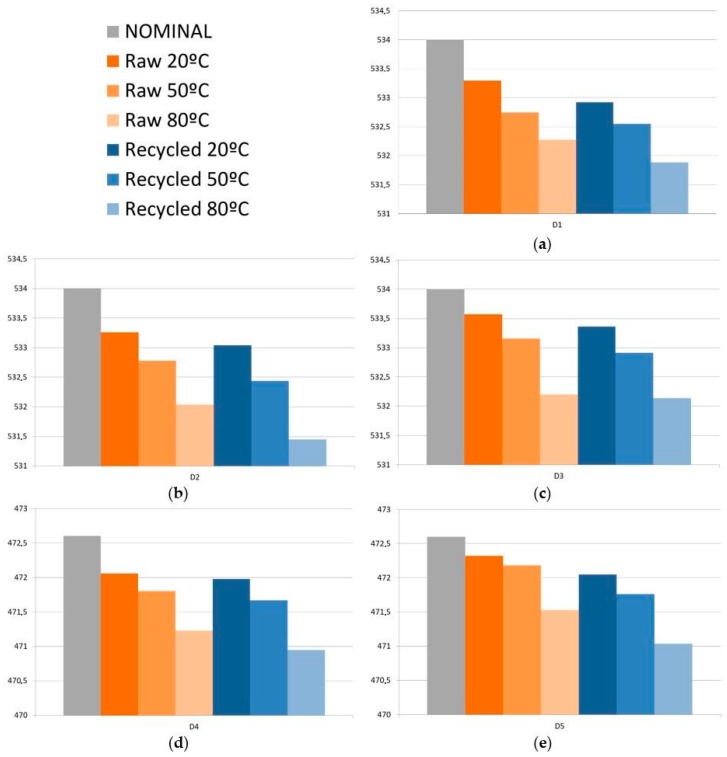
Di measurements (mm) at different environmental conditions for raw and recycled material, (**a**) D1; (**b**) D2; (**c**) D3; (**d**) D4; (**e**) D5.

As previously described, storage temperature conditions are achieved after heating the samples at *M*_1,_ and later stabilizing them at room temperature. Extreme performance conditions are achieved after heating the samples at *M*_2_, and later stabilizing them at room temperature. Figure 5 shows the average dimension values at *M*_1_ and *M*_2_ conditions for all dimensions and each type of material.

All dimensions—D1, D2, D3, D4, and D5—are reduced for both raw and recycled materials when samples are heated above room temperature and then stabilized again at 20 °C. Lower dimensions are achieved when heating them at 80 °C rather than at 50 °C. On the other hand, samples injected with recycled material reach lower dimensions than those injected with raw material for the same heating temperatures. Recycled material is subjected to two different contributions leading to higher deformations, and so, to a lower dimension value. First, higher residual stresses are achieved in recycled material, and second, they tend to have a lower Young Modulus [48,49]. During the injection molding process, residual stresses are stored into samples due to the conformed cooling into the mold cavity until the sample is ejected. When samples are heated again, residual stresses are released, leading to dimensional changes on the sample [50,51]. According to literature, higher residual stresses are stored in a recycled polymer than in raw materials [52]. Thus, higher stresses will be released at higher temperatures for recycled material when recycling PP, changes related to polymer structure are induced. The most relevant modification is the reduction of the length of the polymer chains [53]. The scission of the polymer changes has two main effects that contribute to a Young modulus reduction. First, molecular weight Mw is reduced as polymer chains are shorter [54] and, second, the shorter chains are now included into the amorphous phase of the polymer, leading to a decrease of the Young modulus [48,49].

D1, D2, and D3 keep the same trend in the dimensional variation regarding the nominal value as described for the case, in which samples are stabilized at 20 °C. Higher differences are achieved for D1, and lower for D3. As previously described, non-uniform cooling of the part cavity or differences in stiffness of the cross-section can lead to these results. Dimension D3 is also the one that exhibits a lesser difference between raw and recycled material, especially with samples heated at 80 °C.

### 3.3. Dimensional Behavior after Heating

**Figure 6:**
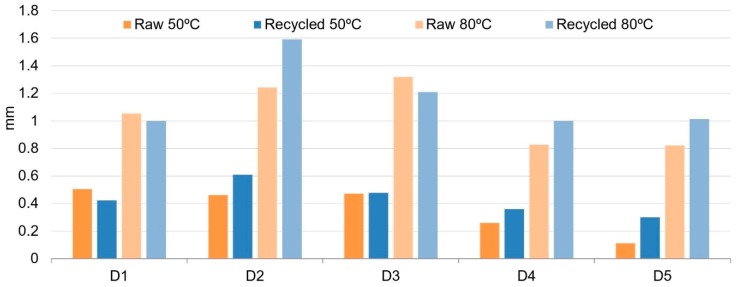
Dimension differences between heated samples and non-heated samples without heating after stabilization at room temperature.

Figure 6 shows dimension differences between heated samples and non-heated samples after stabilization at room temperature, and both obtained from Equations (2) and (3) for raw and recycled material.

These results give us a measure of the shrinkage of these two different materials. As expected, dimension differences for samples heated up to 50 °C are lower than for samples heated up to 80 °C for both materials, raw and recycled. This is due to the higher temperature difference between 80 °C and room temperature that leads to a higher change in the sample volume regarding PVT (pressure-volume-temperature) curves [55]. For samples heated at a temperature of 50 °C, raw material samples exhibit lower differences than the recycled material ones, although for D1 and D3 the difference is very low. For samples heated at a temperature of 80 °C, recycled material samples show higher differences than the raw ones, except for D1 and D3 dimensions, in which recycled material exhibit lower changes regarding non-heated samples, especially in D3.

**Figure 7:**
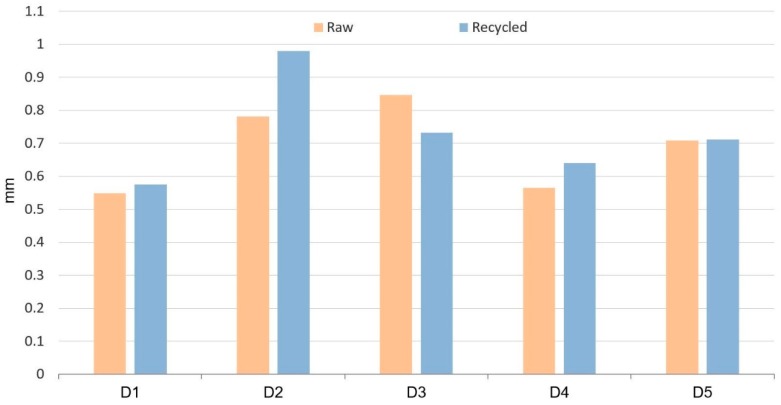
Dimension differences between heated samples at 50 °C and samples heated at 80 °C, after stabilization at room temperature.

Figure 7 shows differences of measures between samples heated at 50 °C and samples heated at 80 °C. This result obtained from Equation (4) gives information about the shrinkage behavior of the samples between two different temperatures but both above room temperature. Again, differences with raw material are lower for all the dimensions, except for D1 and D3.

Residual stresses induced during the injection molding process are released when the parts are heated, leading to distortion and dimensional changes. As the heating temperature is higher (80 °C), released stresses are higher and, thus, higher dimensional changes are observed. This mechanism applies equally to recycled and raw materials. This is shown in Table 3, where dimensional changes induced on samples subjected to a temperature difference of 30 °C are registered. It can be observed that when the temperature difference is applied between 80 and 50 °C (Figure 7), dimensional changes are higher than when the temperature difference is applied between 50 and 20 °C (Figure 6). This trend is observed for all dimensions Di measured.

### 3.4. Process Capability Parameters (C_p_ and C_pk_)

Given that samples manufactured with recycled material achieve lower post-processing dimensions than those injected with raw material, it is required to evaluate if the changes in dimensions are acceptable from a quality point of view. To evaluate the influence of recycled material on the process capability, parameters *C*_p_ and *C*_pk_ have been calculated on Table 4.

It is commonly accepted in the industry that values of 1.33 for *C*_p_ and *C*_pk_ are suitable to consider the process under dimensional control [41]. Therefore, it can be stated that all the dimensions are under the specifications. In the case of raw material, *C*_pk_ values are 2.35 for the D1 dimension and 5.09 for D4 dimension. These values are higher enough to consider that more than 99.9999998% of the samples are within these limits [42].

For recycled material, *C*_pk_ values are 1.704 for D1 dimension and 1.715 for D4 dimension, higher than 1.33, and enough to consider that more than 99.994% of the samples are within limits [42]. Accordingly, with the results obtained in the previous section in which dimensions reduction were higher for samples manufactured with recycled material; *C*_p_ and *C*_pk_ values are lower, and, thus, fewer samples are into the specifications. In any case, the value is higher than 1.33 and, so, high enough to make sure the process capability.

**Figure 8:**
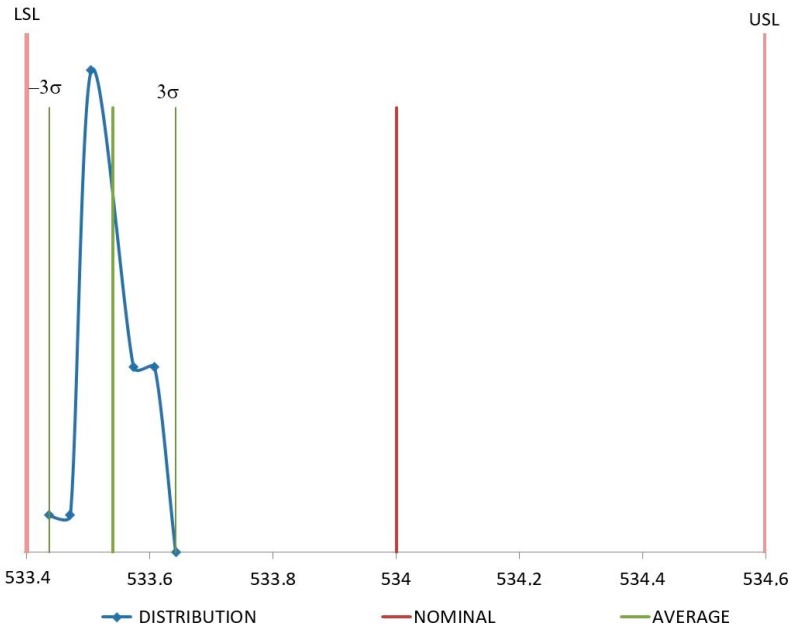
Distribution for D1 (mm) with raw material.

**Figure 9:**
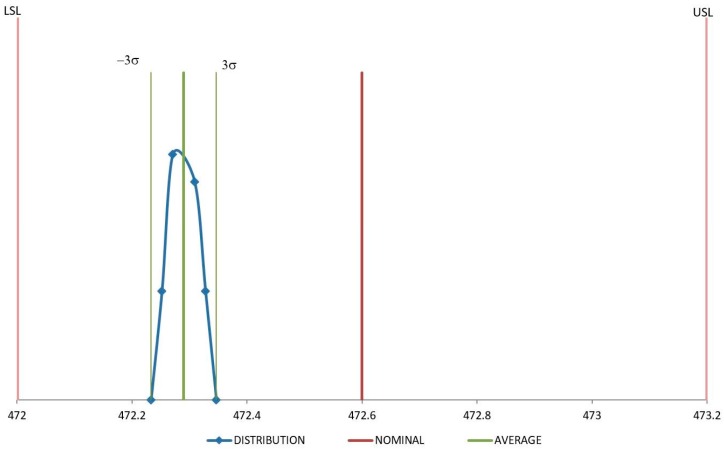
Distribution for D4 (mm) with raw material.

Figure 8 and Figure 9 show distribution of the measures for samples injected with raw material. In both cases these measures are quite close around the average value, although they are not centered on the nominal value, according to previous results of reduction of the dimension values after injection molding due to a volumetric shrinkage of the samples. In any case, both dimensions remain under the specifications.

**Figure 10:**
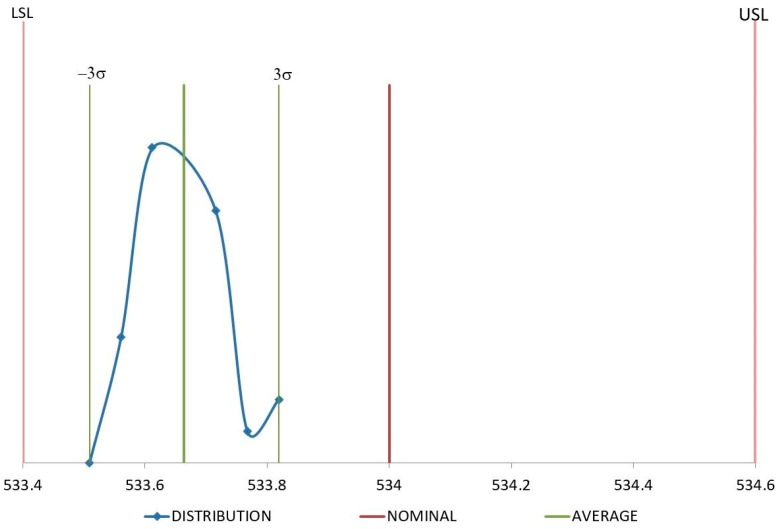
Distribution for D1 (mm) with recycled material.

**Figure 11:**
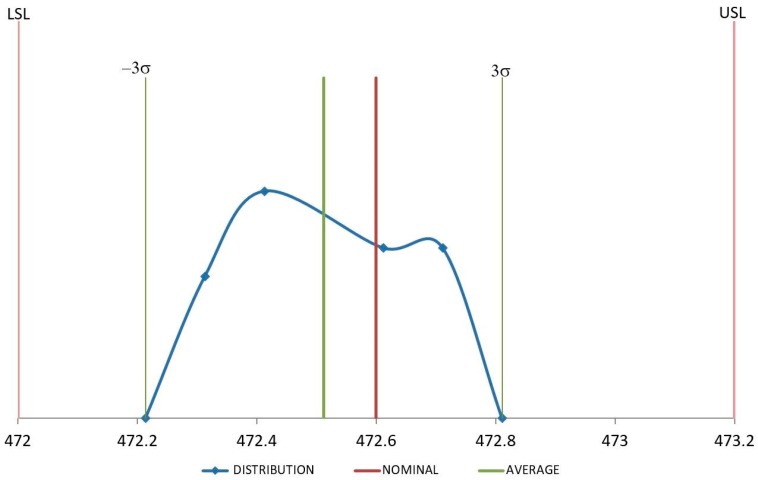
Distribution for D4 (mm) with recycled material.

In the case of recycled material, although *C*_pk_ values are quite similar for D1 and D4, their distributions are not the same as shown in Figure 10 and Figure 11. D1 distribution of values is much more packed around the average value of the samples and less centered on the nominal value. D4 exhibits the opposite trend, values are more dispersed from the average, but they are more centered on the nominal one. Since the value of *C*_pk_ is influenced by both, dispersion and proximity to nominal values [41], *C*_pk_ for D1 and D4 remain similar although their distributions are quite different. Molding tools are usually machined with dimensions over the nominal values to try and compensate the volumetric shrinkage of the component during the cooling stage. Therefore, it is possible to obtain dimension values over and under the nominal value for a set of samples injected under the same conditions just depending on their level of volumetric shrinkage induced by the process. D4 dimension can have an over-dimension in the mold cavity higher than the ones for the D1 dimension. It implies that some of the D4 measures are likely to be higher than the nominal dimension, giving the distribution described in Figure 11. If the overdimension for D1 is lower, it is less probable that D1 measures are over the nominal dimension, as shown in Figure 10.

## 4. Conclusions

A systematic analysis of the dimensional stability of raw and recycled PP under realistic long-term conditions has been carried out. Variations in key dimensions are determined under manufacturing ejection, storage, and extreme performance conditions. In all the cases dimensions are reduced regarding nominal values due to the volumetric shrinkage of the material according to PVT curves. Not all dimensions exhibit the same behavior. Differences between those with the same nominal value are reported and attributed to a differential cooling and stiffness at different sections of the sample. Scissions of the polymer chains provoke lower Young modulus and higher residual stresses, leading to lower dimensions in samples manufactured with recycled material. When samples are subjected to realistic conditions involving high temperatures (80 °C), dimensions are lowered more than for conditions at 50 °C. In all the performed tests, dimensional differences between both materials are small, showing that, for this part, recycled PP can be an alternative to raw PP, without affecting its assembly and functionality.

The processing capability of the manufacturing process is higher than the standard value of 1.33 for both raw and recycled material. In spite of the higher differences regarding the nominal value obtained for samples with recycled material, the process capability is adequate for both materials. At this point, recycled ones arise as an alternative for manufacturing from the point of view of the dimensional stability and process capability.

## Figures and Tables

**Table 1:** General properties of materials.

	Raw	Recycled
Density (g/cm^3^)	1.22	1.25
Vicat B °C 10 N (50 °C/h)	90	94
HDT °C 1.82 MN/m² ISO 75 242 °C	70	74
Molding shrinkage % ISO 294-4	1.35	1.15
Ejection temperature (°C)	110	118

**Table 2:** Measurements (mm) at M_0_ conditions.

	**Raw Material**				
	**D1**	**D2**	**D3**	**D4**	**D5**
Avg *M*_0_	533.29	533.25	533.57	472.05	472.32
Avg ∆*M*_0_	0.72	0.74	0.42	0.54	0.27
σ	0.25	0.05	0.10	0.07	0.26
	**Recycled Material**				
	**D1**	**D2**	**D3**	**D4**	**D5**
Avg *M*_0_	532.92	533.03	533.36	471.97	472.04
Avg ∆*M*_0_	1.07	0.96	0.63	0.62	0.55
σ	0.06	0.06	0.05	0.09	0.07

**Table 3:** Dimensions differences (mm) for ∆T = 20–50 °C and ∆T = 50–80 °C.

	**Raw Material**				
**∆T**	**D1**	**D2**	**D3**	**D4**	**D5**
50–80 °C	0.64	0.78	1.41	0.56	0.7
20–50 °C	0.4	0.46	0.47	0.24	0.11
	**Recycled Material**				
50–80 °C	0.57	0.98	0.73	0.64	0.71
20–50 °C	0.42	0.60	0.47	0.35	0.29

**Table 4:** *C*_p_ and *C*_pk_ for both materials.

	Raw Material		Recycled Material	
	D1	D4	D1	D4
*C*_p_	5.83	10.55	3.873	2.008
*C*_pk_	2.35	5.09	1.704	1.715

## References

[1] Genis A.V. (2017). Analysis of the Global and Russian Markets of Polypropylene and of Its Main Consumption Areas. Russ. J. Gen. Chem..

[2] Hottle T.A., Bilec M.M., Landis A.E. (2017). Biopolymer production and end of life comparisons using life cycle assessment. Resour. Conserv. Recycl..

[3] A European Strategy for Plastics in a Circular Economy European Commission Environment. https://eur-lex.europa.eu/resource.html?uri=cellar:2df5d1d2-fac7-11e7-b8f5-01aa75ed71a1.0001.02/DOC_1&format=PDF.

[4] A European Strategy for Plastics in a Circular Economy (Annexes) European Commission Environment. https://eur-lex.europa.eu/resource.html?uri=cellar:2df5d1d2-fac7-11e7-b8f5-01aa75ed71a1.0001.02/DOC_2&format=PDF.

[5] Trica C.L., Banacu C.S., Busu M. (2019). Environmental Factors and Sustainability of the Circular Economy Model at the European Union Level. Sustainability.

[6] De Mattos C.A., De Albuquerque T.L.M. (2018). Enabling Factors and Strategies for the Transition Toward a Circular Economy (CE). Sustainability.

[7] Cordova-Pizarro D., Aguilar-Barajas I., Romero D., Rodriguez C.A. (2019). Circular Economy in the Electronic Products Sector: Material Flow Analysis and Economic Impact of Cellphone E-Waste in Mexico. Sustainability.

[8] Poponi S., Colantoni A., Cividino S.R., Mosconi E.M. (2019). The Stakeholders’ Perspective within the B Corp Certification for a Circular Approach. Sustainability.

[9] Brown P., Bocken N., Balkenende R. (2019). Why Do Companies Pursue Collaborative Circular Oriented Innovation?. Sustainability.

[10] Woern A.L., Byard D.J., Oakley R.B., Fiedler M.J., Snabes S.L., Pearce J.M. (2018). Fused Particle Fabrication 3-D Printing: Recycled Materials’ Optimization and Mechanical Properties. Materials.

[11] Simões C., Costa Pinto L., Bernardo C. (2013). Environmental and economic assessment of a road-safety product made with virgin and recycled HDPE: A comparative study. J. Environ. Manag..

[12] Rajendran S., Scelsi L., Hodzic A., Soutis C., Al-Maadeed M. (2012). Environmental impact assessment of composites containing recycled plastics. Resour. Conserv. Recycl..

[13] Noda R., Komatsu M., Sumi E., Kasakura T. (2001). Evaluation of material recycling for plastics: Environmental aspects. J. Mater. Cycles Waste Manag..

[14] Vo P.P., Doan H.N., Kinashi K., Sakai W., Tsutsumi N., Huynh D.P. (2018). Centrifugally Spun Recycled PET: Processing and Characterization. Polymers.

[15] Mai V.-D., Shin S.-R., Lee D.-S., Kang I. (2019). Thermal Healing, Reshaping and Eco-friendly Recycling of Epoxy Resin Crosslinked with Schiff Base of Vanillin and Hexane-1,6-Diamine. Polymers.

[16] Galve J.E., Elduque D., Pina C., Javierre C. (2016). Sustainable Supply Chain Management: The Influence of Disposal Scenarios on the Environmental Impact of a 2400 L Waste Container. Sustainability.

[17] Yazdanbakhsh A., Bank L.C. (2014). A Critical Review of Research on Reuse of Mechanically Recycled FRP Production and End-of-Life Waste for Construction. Polymers.

[18] Devasahayam S., Raman R.K.S., Chennakesavulu K., Bhattacharya S. (2019). Plastics—Villain or Hero? Polymers and Recycled Polymers in Mineral and Metallurgical Processing—A Review. Materials.

[19] Achilias D.S., Roupakias C., Megalokomos P., Lappas A.A., Antonakou E.V. (2007). Chemical recycling of plastic wastes made from polyethylene (LDPE and HDPE) and polypropylene (PP). J. Hazard. Mater..

[20] Aurrekoetxea J., Sarrionandia M.A., Urrutibeascoa I., Maspoch M.L. (2001). Effects of recycling on the microstructure and the mechanical properties of isotactic polypropylene. J. Mater. Sci..

[21] Kwon D.E., Park B.K., Lee Y.-W. (2019). Solid-State Foaming of Acrylonitrile-Butadiene-Styrene/Recycled Polyethylene Terephthalate Using Carbon Dioxide as a Blowing Agent. Polymers.

[22] Dal Lago E., Boaretti C., Piovesan F., Roso M., Lorenzetti A., Modesti M. (2019). The Effect of Different Compatibilizers on the Properties of a Post-Industrial PC/PET Blend. Materials.

[23] Samper M.D., Bertomeu D., Arrieta M.P., Ferri J.M., López-Martínez J. (2018). Interference of Biodegradable Plastics in the Polypropylene Recycling Process. Materials.

[24] Javierre C., Clavería I., Ponz L., Aísa J., Fernández A. (2007). Influence of the recycled material percentage on the rheological behaviour of HDPE for injection moulding process. Waste Manag..

[25] Fernandez A., Muniesa M., Javierre C. (2014). In-line rheological testing of thermoplastics and monitored device for an injection moulding machine: Application to a raw and recycled polypropylene. Polym. Test..

[26] Zheng R., Tanner I.R., Fan X. (2011). Shrinkage and warpage. Injection Molding: Integration of Theory and Modeling Methods.

[27] Wang X., Li H., Gu J., Li Z., Ruan S., Shen C., Wang M. (2017). Pressure Analysis of Dynamic Injection Molding and Process Parameter Optimization for Reducing Warpage of Injection Molded Products. Polymers.

[28] Liao S.J., Chang D.Y., Chen H.J., Tsou L.S., Ho J.R., Yau H.T., Hsieh W.H., Wang J.T., Su Y.C. (2004). Optimal process conditions of shrinkage and warpage of thin-wall parts. Polym. Eng. Sci..

[29] Gandhi U., Song Y.Y., Mandapati R. (2017). Semiempirical approach to predict shrinkage and warpage of fiber-reinforced polymers using measured material properties in finite element model. J. Thermoplast. Compos. Mater..

[30] Jiang Q., Liu H., Xiao Q., Chou S., Xiong A., Nie N. (2017). Three-dimensional numerical simulation of total warpage deformation for short-glass-fiber-reinforced polypropylene composite injection-molded parts using coupled FEM. J. Polym. Eng..

[31] Barghash M.A., Alkaabneh F.A. (2014). Shrinkage and Warpage Detailed Analysis and Optimization for the Injection Molding Process Using Multistage Experimental Design. Qual. Eng..

[32] Khanjanzadeh H., Tabarsa T., Shakeri A. (2017). Morphology, dimensional stability and mechanical properties of polypropylene–wood flour composites with and without nanoclay. J. Reinf. Plast. Compos..

[33] De Carvalho Fernandes A., Fagali De Souza A., Leite Howarth J.L. (2016). Mechanical and dimensional characterisation of polypropylene injection moulded parts in epoxy resin/aluminium inserts for rapid tooling. Int. J. Mater. Prod. Technol..

[34] Kusumoto N., Takata K., Kurimoto Y. (2016). Mechanical Properties and Dimensional Stabilities of Wood-Polypropylene Composites Prepared Using Mechanochemically Acetylated Japanese Cedar (*Cryptomeria japonica*) Wood Meal. BioResources.

[35] Li S., Zhao G., Wang J. (2017). A method to improve dimensional accuracy and mechanical properties of injection molded polypropylene parts. J. Polym. Eng..

[36] Castany F.J., Serraller F., Clavería I., Javierre C. (2003). Methodology in gas assisted injection Molding. J. Mater. Process. Technol..

[37] Nian S.C., Wu C.Y., Huang M.S. (2015). Warpage control of thin-walled injection molding using local mold temperatures. Int. Commun. Heat Mass Transf..

[38] Bergeret A., Pires I., Foulc M.P., Abadie B., Ferry L., Crespy A. (2001). The hygrothermal behavior of glass-fibre-reinforced thermoplastic composites: A prediction of the composite lifetime. Polym. Test..

[39] Ozcelik B., Erzurumlu T. (2006). Comparison of the warpage optimization in the plastic injection moulding using ANOVA, neural network model and genetic algorithm. J. Mater. Process. Technol..

[40] Mehat N.M., Kammarudin S. (2011). Optimization of mechanical properties of recycled plastic products via optimal processing parameters using the Taguchi method. J. Mater. Process. Technol..

[41] Kotz S., Johnson N.L. (1993). Process Capability Indices.

[42] Patel S. (2016). The Tactical Guide to Six Sigma Implementation.

[43] Negri Bossi Products. https://www.negribossi.com/wp-content/uploads/VECTOR-depliant_Negri-Bossi_2016-10-3-1.pdf.

[44] Claveria I., Elduque D., Santolaria J., Pina C., Javierre C., Fernández A. (2016). The influence of environmental conditions on the dimensional stability of components injected with PA6 and PA66. Polym. Test..

[45] Zeiss Coordinate Measuring Machines. https://www.zeiss.com/metrology/products/systems/coordinate-measuring-machines.html.

[46] Zeiss Tactile Scanning Probes. https://www.zeiss.com/metrology/products/sensors/on-cmm/tactile-scanning-probe.html.

[47] Zeiss Calypso—Measuring Software for Geometry. https://www.zeiss.com/metrology/products/software/calypso-overview/calypso.html.

[48] Rogueda-Berriet C., Bahlouli N., Pessey D., Rémond Y. (2011). Mechanical Behavior of Recycled Polypropylene Composites Under Tensile, Bending, and Creep Loading: Experimental and Modeling. J. Eng. Mater. Technol..

[49] Incarnato L., Scarfato P., Acierno D. (1999). Rheological and mechanical properties of recycled polypropylene. Pol. Eng. Sci..

[50] Katmer S., Esen H., Kataras C. (2016). Residual stresses in injection molded shape memory polymer parts. Proceedings of the 31st International Conference of the Polymer Processing Society.

[51] Kallel A., Lamraoui M., Fitoussi J., Tcharkhtchi A. (2016). The Residual Stress Effect on the Shape Memory Polymers. Mater. Res. Proc..

[52] Altan M., Yurci M.E., Nugay M. (2008). Residual stresses determination in injection molded virgin and recycled HDPE blends: Mechanical properties and morphology. e-Polymers.

[53] Valenza A., La Mantia F.P. (1988). Recycling of polymer waste: Part II—Stress degraded polypropylene. Polym. Degrad. Stab..

[54] González-González V.A., Neira-Velázquez G., Angulo-Sánchez J.L. (1998). Polypropylene chain scissions and molecular weight changes in multiple extrusion. Polym. Degrad. Stab..

[55] Sun X., Su X., Tibberham P., Mao J., Tao J. (2016). The application of modified PVT data on the warpage prediction of injection molded part. J. Pol. Res..

